# Clinical significance of protocadherin 8 (PCDH8) promoter methylation in non-muscle invasive bladder cancer

**DOI:** 10.1186/s13046-014-0068-7

**Published:** 2014-08-22

**Authors:** Ying-Li Lin, Yan-Ling Wang, Jian-Guo Ma, Wen-Ping Li

**Affiliations:** 1 Department of Urology, Affiliated Xuzhou Hospital of Jiangsu University (Xuzhou Cancer Hospital), Xuzhou 221000, China; 2 Department of Anesthesiology, Third Affiliated Hospital of Sun Yat-sen University, Guangzhou 510630, China; 3 Department of Urology, Third Hospital of Hebei Medical University, No. 139 Ziqiang Road, Shijiazhuang 050051, Hebei Province, China

**Keywords:** Bladder cancer, Methylation, PCDH8, Protocadherin-8, Biomarker

## Abstract

**Background:**

PCDH8 is a novel tumor suppressor gene, and frequently inactivated by promoter methylation in human cancers. However, there is little information regarding PCDH8 methylation in non-muscle invasive bladder cancer (NMIBC). The aim of this study was to investigate the methylation status of PCDH8 in NMIBC and its clinical significance.

**Methods:**

The methylation status of PCDH8 in 233 NMIBC tissues and 43 normal bladder epithelial tissues was examined by methylation-specific PCR (MSP), and then analyzed the correlations between PCDH8 methylation and clinicopatholocial features. Subsequently, Kaplan-Meier survival analysis and Multivariate Cox proportional hazard model analysis was used to investigate the correlation between PCDH8 methylation and prognosis of patients with NMIBC.

**Results:**

PCDH8 methylation occurred frequently in NMIBC tissues than those in normal bladder epithelial tissues. In addition, PCDH8 methylation significantly correlated with advanced stage, high grade, larger tumor size, tumor recurrence and progression in NMIBC. Kaplan-Meier survival analysis revealed that patients with PCDH8 methylated have shorter recurrence-free survival, progression-free survival and five-year overall survival than patients with PCDH8 unmethylated. Multivariate analysis suggested that PCDH8 methylation was an independent prognostic biomarker for recurrence-free survival, progression-free survival and five-year overall survival simultaneously.

**Conclusions:**

PCDH8 methylation may be associated with tumor progression and poor prognosis in NMIBC and may be used as a potential biomarker to predict the prognosis of patients with NMIBC.

## Background

Bladder cancer is one of the most frequently diagnosed malignancies and a common cause of cancer related death in the human, which has become a major public health problem in the world [1]-[4]. Although most of the newly diagnosed bladder tumors are non-muscle invasive bladder cancer (NMIBC), the majority of these NMIBC cases will relapse after curative transurethral resection, and some will progress to muscle invasive disease ineluctably [5],[6]. Unfortunately, the outcome of bladder cancer is worse with tumor progression [2]. Currently, conventional clinicopathological factors are insufficient to predict the outcome of all the patients with NMIBC accurately. Therefore, new markers are needed to predict the course of NMIBC, which may be helpful in the making of treatment strategies [7]-[10]. As most of other human cancers, the initiation and progression of bladder cancer associates with the accumulation of genetic and epigenetic changes; DNA methylation is the most common and best-characterized epigenetic change in bladder cancer, which inactivates tumor suppressor genes and may be used as potential biomarker [9],[10].

PCDH8 is a member of protocadherin subfamily, which belongs to cadherin super-family [11]-[16]. The protocadherins commonly have six ertracellular cadherin domains, a transmembrane domain, and different cytoplasmic domains. The protocadherins play important roles not only in cell-cell adhesion, but also in signal transduction, growth control, and some of them have tumor-suppressive functions [11]-[16]. In recent years, studies reported that protocadheins often inactivated by promoter methylation in human cancers, and the aberrant promoter methylation can be used as potential biomarker for tumor diagnosis, surveillance, or prognosis, such as PCDH8, PCDH10, PCDH17, and PCDH20, which are considered as tumor suppressor genes in tumors [11]-[21]. Recently, the inactivation of PCDH8 caused by promoter methylation has been reported in human cancers, including bladder cancer [13]-[16]. In our previous study, we found that PCDH8 promoter methylation occurs frequently in bladder cancer, and associates with poor outcomes of bladder cancer patients [13]. However, our previous study included both non-muscle invasive and muscle invasive disease, and the clinical significance of PCDH8 promoter methylation in NMIBC remains largely unclear.

In the present study, the methylation status of PCDH8 in NMIBC and normal bladder epithelial tissues was examined using MSP. Then we investigated the correlation between PCDH8 methylation status and clinicopathologic parameters in NMIBC cases. Moreover, we assessed the influence of PCDH8 methylation on the outcomes of NMIBC patients to evaluate its clinical significance.

## Materials and methods

### Patient tissue specimens

A total of 233 patients with bladder cancer who had a transurethral resection of bladder tumor between January 2004 and January 2008 at the Third Hospital of Hebei Medical University were recruited. All patients were histopathologically diagnosed as non-muscle invasive bladder transitional cell carcinoma for the first time, and they did not receive preoperative anti-cancer therapy. In addition, the normal bladder epithelial tissues obtained from 43 inpatients with bladder stone were also collected as controls; these samples were examined pathologically to exclude the possibility of incidental tumors. The tissue samples were immediately frozen in liquid nitrogen after resection and stored at -80°C until examined. The bladder cancers were graded and staged according to 1973 WHO grading system and 2002 TNM classification [22],[23]. Tumor therapy and follow up strategies were performed according to international guidelines [22]-[24] Recurrence was defined as a new tumor observed in the bladder after initial curative resection and progression was defined as a disease with a higher TNM stage when relapsed [25]. Follow-up continued until the death of the patient or to 60 months if the patient remained alive. This study was approved by the ethics committee of Third Hospital of Hebei Medical University, and written informed consent was obtained from all of the participants.

### DNA extraction, bisulfite modification and MSP

Genomic DNA from the tissue samples was extracted using DNeasy Tissue Kit (Qiagen, Valencia, CA) following the manufacture’s instructions. The quality of extracted DNA was assessed using NanoDrop ND-1000 (Thermo Fisher Scientific, Waltham, USA). The extracted DNA was treated with bisulfite using EpiTect Bisulfite Kit (Qiagen, Valencia, CA) according to the manufacture’s protocol. The bisufite modifited DNA was then used for MSP. The methylation status of PCDH8 was detected using primers specific for PCDH8 unmethylated and methylated sequences respectively, as our reported previously [18]. The following primers were used: unmethylated: forward 5’- GGTGGTTATTGGTTATTTGGTTT-3’ and reverse 5’- CCAACAAACTCTAAAAACACACA-3’; methylated: forward 5’- CGGTTATTGGTTATTCGGTTCC-3’ and reverse 5’- ACGAACTCTAAAAACGCGCG -3’. The PCR amplification of the modified DNA consisted of one cycle of 95°C for 5 min, 40 cycles of 95°C for 30 s, 60°C for 30 s, and 72°C for 30 s, and 1 cycle of 72°C for 5 min. Water blanks were included with each assay, in vitro methylated DNA and unmethylated DNA (New England Biolabs, Beverly, MA, USA) was used as methylation and unmethylation positive control. PCR products were separated in 2% agarose gel, stained with ethidium bromide, and visualized under ultraviolet illumination for analysis. Samples were scored as methylation positive when methylated alleles were present in the methylated DNA lane and methylation negative when bands were present only in the unmethylated DNA lane [18].

### Statistical analysis

Statistical analysis was conducted using SAS version 8.0 (SAS Institute, Cary, N.C., USA). Fisher’s exact test was used to assess the difference of PCDH8 methylation status between NMIBC patients and controls. Chi-square test was used to assess the relationship between PCDH8 methylation and clinicopathologic features. Kaplan-Meier survival analysis and log-rank test were used to assess the differences of recurrence-free survival, progression-free survival and five-year overall survival between patients with PCDH8 methylated and unmethylated. Multivariate Cox proportional hazard model analysis was used to assess the independent prognostic effect of PCDH8 methylation. A two-sided p value < 0.05 was considered statistically significant.

## Results

### The methylation status of PCDH8 in NMIBC and normal bladder epithelial tissues

**Figure 1:**
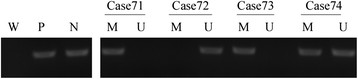
**Representative MSP results for PCDH8 methylation in tumor-derived DNA samples from patients with NMIBC.** W: water; P: positive control; N: negative control; M: methylated; U: unmethylated. Cases 71, 73 and 74 exhibited PCDH8 methylation. Case 72 exhibited PCDH8 unmethylation.

In the current study, the methylation status of PCDH8 in NMIBC and normal bladder epithelial tissues was examined by MSP. We found that PCDH8 methylation occurred in 128 (54.9%) patients with NMIBC (Figure 1). However, no methylation was detected in controls, and the difference between these two groups was statistically significant. The result is shown in Table 1.

**Table 1:** The methylation status of PCDH8 in NMIBC and normal bladder epithelial (NBE) tissues

**Group**	**M (%)**	**U (%)**	**P**
NMIBC	128 (54.9)	105 (45.1)	<0.0001
NBE	0 (0.0)	43 (100.0)	

*M*: Methylation; *U*: Unmethylation.

### Relationship between PCDH8 methylation and clinicopathological characteristics

The main purpose of this study was to investigate the clinical significance of PCDH8 methylation in NMIBC, so we investigated the relationships between PCDH8 methylation and clinicopathological features in bladder cancer. The association of PCDH8 methylation with the clinicopathological features is summarized in Table 2. The promoter methylation of PCDH8 in NMIBC tissues was correlated with, advanced stage (P = 0.0138), high grade (P = 0.0010), larger tumor size (P = 0.0482), tumor recurrence (P < 0.0001) and tumor progression (P < 0.0001) significantly. However, the promoter methylation of PCDH8 was not correlated with age, gender, and tumor number.

**Table 2:** Relationship between PCDH8 methylation and clinicopathological characteristics in NMIBC (n = 233)

**Features**	**Variables**	**No.**	**M (%)**	**U (%)**	**P**
Age	65	86	46(53.5)	40(46.5)	0.7342
	>65	147	82(55.8)	65(44.2)	
Sex	Male	161	94(58.4)	67(41.6)	0.1135
	Female	72	34(47.2)	38(52.8)	
Number	Single	142	82(57.8)	60(42.2)	0.2814
	Multiple	91	46(50.6)	45(49.4)	
Size	≤3 cm	139	69(49.6)	70(50.4)	0.0482
	>3 cm	94	59(62.8)	35(37.2)	
Grade	G_1_/ G_2_	144	67(46.5)	77(53.5)	0.0010
	G_3_	89	61(68.5)	28(31.5)	
Stage	T_a_	95	43(45.3)	52(54.7)	0.0138
	T_1_	138	85(61.6)	53(38.4)	
Recurrence	No	127	40(31.5)	87(68.5)	<0.0001
	Yes	106	88(83.0)	18(17.0)	
Progression	No	175	80(45.7)	95(54.3)	<0.0001
	Yes	58	48(82.8)	10(17.2)	

*M*: Methylation; *U*: Unmethylation.

### The impact of PCDH8 methylation on the clinical outcome of NMIBC

**Figure 2:**
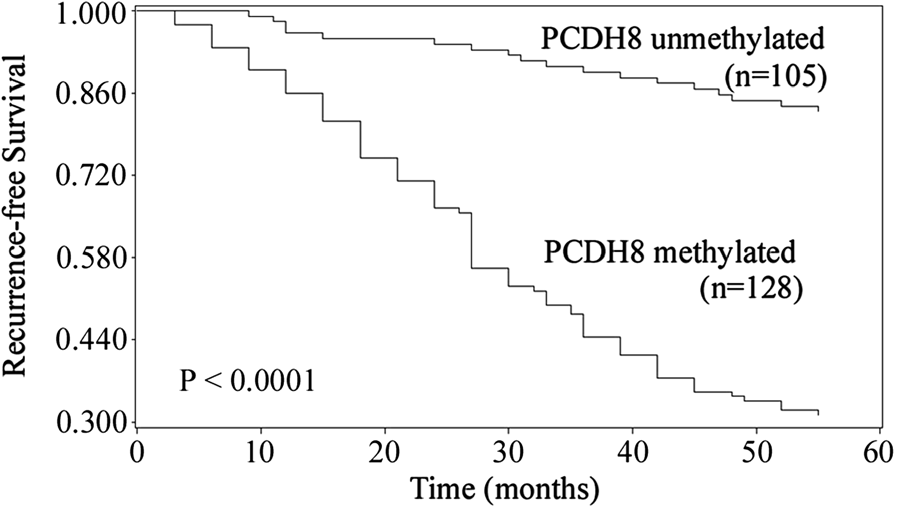
**Correlations between PCDH8 methylation and recurrence-free survival in NMIBC patients.** Patients with PCDH8 methylated showed significantly shorter recurrence-free survival than patients without (P < 0.0001, log-rank test).

**Figure 3:**
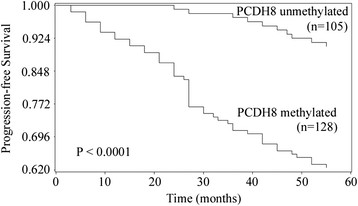
**Correlations between PCDH8 methylation and progression-free survival in NMIBC patients.** Patients with PCDH8 methylated showed significantly shorter progression-free survival than patients without (P < 0.0001, log-rank test).

**Figure 4:**
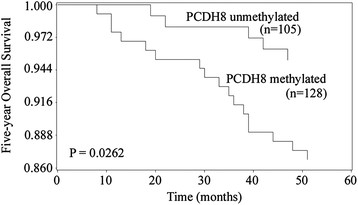
**Correlations between PCDH8 methylation and five-year overall survival in NMIBC patients.** Patients with PCDH8 methylated showed significantly shorter five-year overall survival than patients without (P = 0.0177, log-rank test).

To examine if PCDH8 promoter methylation is a potential predictor of the prognosis in NMIBC, the recurrence-free survival, progression-free survival and five-year overall survival was analyzed, and the NMIBC patients was divided into two subgroup according to PCDH8 methylation status. Kaplan-Meier survival analysis and log-rank test suggested that NMIBC patients with PCDH8 methylated had significantly shorter recurrence-free survival (P < 0.0001; Figure 2), progression-free survival (P < 0.0001; Figure 3) and five-year overall survival (P = 0.0262; Figure 4) than patients with PCDH8 unmethylaed respectively. Moreover, multivariate Cox proportional hazard model analysis indicated that PCDH8 promoter methylation in tissues was an independent predictor of shorter recurrence-free survival (P < 0.0001; Table 3), progression-free survival (P =0.0036; Table 4) and five-year overall survival (P = 0.0015; Table 5).

**Table 3:** The predictive value of PCDH8 methylation for the recurrence-free survival in non muscle invasive bladder cancer (n = 233)

**Variable**	**Univariate analysis**			**Multivariate analysis**		
	**HR**	**95% CI**	**P**	**HR**	**95% CI**	**P**
PCDH8 methylation (M vs. U)	7.083	2.576-14.621	<0.0001	4.739	1.872-12.053	<0.0001
Age (>65 vs. ≦65)	1.241	0.768-5.724	0.7931			
Sex (Male vs. Female)	0.926	0.753-3.761	0.8541			
Number (Multiple vs. Single)	1.411	0.674-12.653	0.7244			
Size (>3 cm vs. ≤3 cm)	1.537	0.687-10.431	0.7196			
Grade (G_3_ vs. G_1_/G_2_)	5.067	1.933-10.763	0.0006	2.055	1.644-8.431	0.0137
Stage (T_1_ vs. T_a_)	2.073	1.027-9.754	0.0176	1.371	0.824-6.084	0.0735

*HR*: Hazard Ratio; *M*: Methylated; *U*: unmethylated.

**Table 4:** The predictive value of PCDH8 methylation for the progression-free survival in non muscle invasive bladder cancer (n = 233)

**Variable**	**Univariate analysis**			**Multivariate analysis**		
	**HR**	**95% CI**	**P**	**HR**	**95% CI**	**P**
PCDH8 methylation (M vs. U)	4.893	1.872-9.433	<0.0001	2.523	1.654-7.431	0.0036
Age (>65 vs. ≦65)	0.896	0.873-5.215	0.8614			
Sex (Male vs. Female)	1.213	0.855-5.217	0.5461			
Number (Multiple vs. Single)	1.322	0.729-8.537	0.4668			
Size (>3 cm vs. ≤3 cm)	1.227	0.579-11.460	0.4962			
Grade (G_3_ vs. G_1_ / G_2_)	3.679	1.463-7.754	0.0017	1.874	1.237-6.873	0.0233
Stage (T_1_ vs. T_a_)	1.625	0.893-6.792	0.0614			

*HR*: Hazard Ratio; *M*: Methylated; *U*: Unmethylated.

**Table 5:** The predictive value of PCDH8 methylation for the five-year overall survival in non muscle invasive bladder cancer (n = 233)

**Variable**	**Univariate analysis**			**Multivariate analysis**		
	**HR**	**95% CI**	**P**	**HR**	**95% CI**	**P**
PCDH8 methylation (M vs. U)	4.653	1.237-7.314	<0.0001	3.017	1.542-8.251	0.0015
Age (>65 vs. ≦65)	1.135	0.779-6.273	0.3471			
Sex (Male vs. Female)	0.874	0.645-3.228	0.7361			
Number (Multiple vs. Single)	1.054	0.798-6.417	0.3784			
Size (>3 cm vs. ≤3 cm)	1.253	0.913-10.257	0.3095			
Grade (G_3_ vs. G_1_ / G_2_)	3.876	1.643-6.024	0.0021	1.852	1.144-5.964	0.0324
Stage (T_1_ vs. T_a_)	1.015	0.792-7.572	0.4338			

*HR*: Hazard Ratio; *M*: Methylated; *U*: Unmethylated.

## Discussion

Bladder cancer is a multifaceted disease with clinical outcome difficult to predict, and the morphological similar tumors can behave differently [2]. Thus, new biomarkers are needed to predict the outcome of bladder cancer, in addition to commonly used clinicopathological parameters [2]. In recent years, more and more researchers are interested in the aberrant methylation of different genes in bladder cancer for some reasons [9],[10],[26]. Firstly, aberrant methylation in the promoter regions of the tumor suppressor genes at CPG islands has been recognized as one of the hallmarks of human cancers and associated with silence of gene expression, which may be used as potential biomarker in human cancers [27]-[31]. Secondly, DNA methylation can be reversed by demethylating agents, which may used as effective therapeutic target. PCDH8 is a novel tumor suppressor gene, and commonly inactivated by aberrant promoter methylation in human cancers [11]-[16]. In the current study, we are focused on the methylation of PCDH8 in NMIBC, and clarified its clinical significance.

In this study, the methylation status of PCDH8 in NMIBC tissues and normal bladder epithelial tissues was examined by MSP. MSP is a rapid, simple, sensitive, specific, cost effective method for methylation detection, and allowing the rapid examination of multiple samples, which is convenient for routine clinical use [32],[33]. We found that PCDH8 methylation occurred frequently in NMIBC tissues, while no methylation was detected in normal bladder epithelial tissues. This finding indicated that PCDH8 methylation is tumor specific, may be involved in the tumorigenesis of bladder cancer, and giving the possibility to investigate its clinical significance in NMIBC. Subsequently, we investigated the associations between PCDH8 methylation and clinicopathological factors in NMIBC cases only. PCDH8 methylation was significantly associated with higher grade, advanced stage, larger tumor size, and multiple tumor number. These factors are considered as risk factors for the progression of bladder cancer [2]-[5]. Therefore, PCDH8 may be involved in the progression of NMIBC. Amazingly, when we correlated PCDH8 methylation to the recurrence and progression of NMIBC, we found that PCDH8 methylation significantly associated with the recurrence and progression of NMIBC after initial adequate treatment. Our data suggested that PCDH8 methylation may be correlated with poor outcome of patients with NMIBC, and may be a potential predictive biomarker for the prognosis.

To further investigate the prognostic value of PCDH8 methylation in NMIBC, the recurrence-free survival, progression-free survival and five-year overall survival was analyzed according to the methylation status of PCDH8 in tumor samples. Kaplan-Meier survival analysis and log-rank test demonstrated that patients with PCDH8 methylation had significantly unfavorable recurrence-free survival, progression-free survival and five-year overall survival than patients with PCDH8 unmethylated. Moreover, multivariate Cox proportional hazard model analysis indicated that PCDH8 methylation was an independent prognostic biomarker for recurrence-free survival, progression-free survival and five-year overall survival simultaneously. These results indicate that PCDH8 methylation plays an important role in the initiation and progression of NMIBC, is significantly correlated with poor prognosis independently.

Furthermore, the significant role of PCDH8 methylation in NMIBC indicates the possibility to make it as a potential therapeutic target. Previous studies have revealed that the methylation status of PCDH8 in tumor cell lines can be reversed by demethylating agents and restore PCDH8 expression. The restoration of PCDH8 expression plays crucial role in the inhabitation of tumor cell proliferation, migration and invasion, which are all crucial factors of tumor progression [14]-[16]. Taken together, these findings suggest the possibility to make PCDH8 methylation a potential target for anticancer therapy in NMIBC.

## Conclusions

In conclusion, PCDH8 methylation occurred frequently in NMIBC, and correlated higher grade, advanced stage, larger tumor size, tumor recurrence and progression. Moreover, PCDH8 methylation was an independent prognostic biomarker for recurrence-free survival, progression-free survival and five-year overall survival simultaneously. Thus for NMIBC patients with PCDH8 methylated in tumor samples after initial transurethral resection of primary tumor more aggressive adjunctive therapy should be considered, in order to achieve better prognosis. In addition, PCDH8 methylation may be used as an effective therapeutic target in NMIBC. However, our study was limited by relative small sample size in mono-center, and future studies with larger sample size in multiple centers are needed to confirm our findings before used routinely in clinical practice.

## Competing interests

The authors declare that they have no competing interests.

## Authors’ contribution

LYL and WYL carried out the molecular studies, statistical analysis, data collection and data interpretation; MJG and LWP involved in study design, manuscript preparation, literature search and funds collection. LYL and WYL co-first author. All authors read and approved the final manuscript.

## References

[B1] Siegel R1 Naishadham D Jemal A Cancer statistics, 2013 CA Cancer J Clin 2013 63 1 11 30 10.3322/caac.21166 23335087

[B2] Kaufman DS Shipley WU Feldman AS Bladder cancer Lancet 2009 374 9685 239 249 10.1016/S0140-6736(09)60491-8 19520422

[B3] Parkin DM The global burden of urinary bladder cancer Scand J Urol Nephrol Suppl 2008 218 12 20 10.1080/03008880802285032 19054893

[B4] Ploeg M Aben KK Kiemeney LA The present and future burden of urinary bladder cancer in the world World J Urol 2009 27 3 289 293 10.1007/s00345-009-0383-3 19219610 PMC2694323

[B5] Van den Bosch S Alfred Witjes J Long-term cancer-specific survival in patients with high-risk, non-muscle-invasive bladder cancer and tumour progression: a systematic review Eur Urol 2011 60 3 493 500 10.1016/j.eururo.2011.05.045 21664041

[B6] Van Rhijn BW Burger M Lotan Y Solsona E Stief CG Sylvester RJ Witjes JA Zlotta AR Recurrence and progression of disease in non-muscle-invasive bladder cancer: from epidemiology to treatment strategy Eur Urol 2009 56 3 430 442 10.1016/j.eururo.2009.06.028 19576682

[B7] Musquera M Mengual L Ribal MJ Non-invasive diagnosis bladder cancer: new molecular markers and future perspectives Arch Esp Urol 2013 66 5 487 494 23793766

[B8] Galustian C Tools to investigate biomarker expression in bladder cancer progression BJU Int 2013 112 3 404 406 10.1111/j.1464-410X.2013.11792.x 23826845

[B9] Kandimalla R van Tilborg AA Zwarthoff EC DNA methylation-based biomarkers in bladder cancer Nat Rev Urol 2013 10 6 327 335 10.1038/nrurol.2013.89 23628807

[B10] Kim WJ Kim YJ Epigenetics of bladder cancer Methods Mol Biol 2012 863 111 118 10.1007/978-1-61779-612-8_6 22359289

[B11] Kim SY Yasuda S Tanaka H Yamagata K Kim H Non-clustered protocadherin Cell Adh Migr 2011 5 2 97 105 10.4161/cam.5.2.14374 21173574 PMC3084973

[B12] Chen WV Maniatis T Clustered protocadherins Development 2013 140 16 3297 3302 10.1242/dev.090621 23900538 PMC3737714

[B13] Lin YL Ma JH Luo XL Guan TY Li ZG Clinical significance of protocadherin-8 (PCDH8) promoter methylation in bladder cancer J Int Med Res 2013 41 1 48 54 10.1177/0300060513475571 23569129

[B14] Zhang D Zhao W Liao X Bi T Li H Che X Frequent silencing of protocadherin 8 by promoter methylation, a candidate tumor suppressor for human gastric cancer Oncol Rep 2012 28 5 1785 1791 22941331 10.3892/or.2012.1997

[B15] Yu JS Koujak S Nagase S Li CM Su T Wang X Keniry M Memeo L Rojtman A Mansukhani M Hibshoosh H Tycko B Parsons R PCDH8, the human homolog of PAPC, is a candidate tumor suppressor of breast cancer Oncogene 2008 27 34 4657 4665 10.1038/onc.2008.101 18408767 PMC3013056

[B16] He D Zeng Q Ren G Xiang T Qian Y Hu Q Zhu J Hong S Hu G Protocadherin8 is a functional tumor suppressor frequently inactivated by promoter methylation in nasopharyngeal carcinoma Eur J Cancer Prev 2012 21 6 569 575 10.1097/CEJ.0b013e328350b097 22273848

[B17] Tang X Yin X Xiang T Li H Li F Chen L Ren G Protocadherin 10 is frequently downregulated by promoter methylation and functions as a tumor suppressor gene in non-small cell lung cancer Cancer Biomark 2013 12 1 11 19 23321465 10.3233/CBM-2012-00280 PMC13016365

[B18] Lin YL Li ZG He ZK Guan TY Ma JG Clinical and prognostic significance of protocadherin-10 (PCDH10) promoter methylation in bladder cancer J Int Med Res 2012 40 6 2117 2123 10.1177/030006051204000609 23321168

[B19] Costa VL Henrique R Danielsen SA Eknaes M Patrício P Morais A Oliveira J Lothe RA Teixeira MR Lind GE Jerónimo C TCF21 and PCDH17 methylation: an innovative panel of biomarkers for a simultaneous detection of urological cancers Epigenetics 2011 6 9 1120 1130 10.4161/epi.6.9.16376 21847011

[B20] Yang Y Liu J Li X Li JC PCDH17 gene promoter demethylation and cell cycle arrest by genistein in gastric cancer Histol Histopathol 2012 27 2 217 224 22207556 10.14670/HH-27.217

[B21] Imoto I Izumi H Yokoi S Hosoda H Shibata T Hosoda F Ohki M Hirohashi S Inazawa J Frequent silencing of the candidate tumor suppressor PCDH20 by epigenetic mechanism in non-small-cell lung cancers Cancer Res 2006 66 9 4617 4626 10.1158/0008-5472.CAN-05-4437 16651412

[B22] Greene FL The American Joint Committee on Cancer: updating the strategies in cancer staging Bull Am Coll Surg 2002 87 7 13 15 17387902

[B23] Oosterlinck W Lobel B Jakse G Malmström PU Stöckle M Sternberg C Guidelines on bladder cancer Eur Urol 2002 41 2 105 112 10.1016/S0302-2838(01)00026-4 12074395

[B24] Babjuk M Oosterlinck W Sylvester R Kaasinen E Böhle A Palou-Redorta J Rouprêt M EAU guidelines on non-muscle-invasive urothelial carcinoma of the bladder, the 2011 update Eur Urol 2011 59 6 997 1008 10.1016/j.eururo.2011.03.017 21458150

[B25] Li H Wang J Xiao W Xia D Lang B Wang T Guo X Hu Z Ye Z Xu H Epigenetic inactivation of KLF4 is associated with urothelial cancer progression and early recurrence J Urol 2014 191 2 493 501 10.1016/j.juro.2013.08.087 24018236

[B26] Casadio V Molinari C Calistri D Tebaldi M Gunelli R Serra L Falcini F Zingaretti C Silvestrini R Amadori D Zoli W DNA Methylation profiles as predictors of recurrence in non muscle invasive bladder cancer: an MS-MLPA approach J Exp Clin Cancer Res 2013 32 1 94 10.1186/1756-9966-32-94 24252461 PMC4176288

[B27] Zhu J Wang Y Duan J Bai H Wang Z Wei L Zhao J Zhuo M Wang S Yang L An T Wu M Wang J DNA Methylation status of Wnt antagonist SFRP5 can predict the response to the EGFR-tyrosine kinase inhibitor therapy in non-small cell lung cancer J Exp Clin Cancer Res 2012 31 80 10.1186/1756-9966-31-80 23009178 PMC3524045

[B28] Wang N Zhang H Yao Q Wang Y Dai S Yang X TGFBI promoter hypermethylation correlating with paclitaxel chemoresistance in ovarian cancer J Exp Clin Cancer Res 2012 31 6 10.1186/1756-9966-31-6 22248469 PMC3283468

[B29] Rengucci C De Maio G Gardini A Zucca M Scarpi E Zingaretti C Foschi G Tumedei M Molinari C Saragoni L Puccetti M Amadori D Zoli W Calistri D Promoter methylation of tumor suppressor genes in pre-neoplastic lesions; potential marker of disease recurrence J Exp Clin Cancer Res 2014 33 1 65 10.1186/s13046-014-0065-x 25091577 PMC4274757

[B30] Moon JW Lee SK Lee JO Kim N Lee YW Kim SJ Kang HJ Kim J Kim HS Park SH Identification of novel hypermethylated genes and demethylating effect of vincristine in colorectal cancer J Exp Clin Cancer Res 2014 33 4 10.1186/1756-9966-33-4 24393480 PMC3923411

[B31] Cui X Zhao Z Liu D Guo T Li S Hu J Liu C Yang L Cao Y Jiang J Liang W Liu W Li S Wang L Wang L Gu W Wu C Chen Y Li F Inactivation of miR-34a by aberrant CpG methylation in Kazakh patients with esophageal carcinoma J Exp Clin Cancer Res 2014 33 20 10.1186/1756-9966-33-20 24528540 PMC3931274

[B32] Herman JG Graff JR Myöhänen S Nelkin BD Baylin SB Methylation-specific PCR: a novel PCR assay for methylation status of CpG islands Proc Natl Acad Sci U S A 1996 93 18 9821 9826 10.1073/pnas.93.18.9821 8790415 PMC38513

[B33] Kinoshita K Minagawa M Takatani T Takatani R Ohashi M Kohno Y Establishment of diagnosis by bisulfite-treated methylation-specific PCR method and analysis of clinical characteristics of pseudohypoparathyroidism type 1b Endocr J 2011 58 10 879 887 10.1507/endocrj.K10E-364 21836370

